# Pairwise interaction Markov model for 3D epidermal nerve fibre endings

**DOI:** 10.1111/jmi.13142

**Published:** 2022-09-26

**Authors:** Konstantinos Konstantinou, Aila Särkkä

**Affiliations:** ^1^ Chalmers tekniska hogskola Gothenburg Sweden; ^2^ Department of Mathematical Sciences Chalmers University of Technology Gothenburg Sweden

**Keywords:** anisotropy, cylindrical *K* function, Markov chain Monte Carlo, Markov random field, point process, pseudo‐likelihood

## Abstract

In this paper, the spatial arrangement and possible interactions between epidermal nerve fibre endings are investigated and modelled by using confocal microscopy data. We are especially interested in possible differences between patterns from healthy volunteers and patients suffering from mild diabetic neuropathy. The locations of the points, where nerves enter the epidermis, the first branching points and the points where the nerve fibres terminate, are regarded as realizations of spatial point processes. We propose an anisotropic point process model for the locations of the nerve fibre endings in three dimensions, where the points interact in cylindrical regions. First, the locations of end points in $R^{2}$ are modelled as clusters around the branching points and then, the model is extended to three dimensions using a pairwise interaction Markov field model with cylindrical neighbourhood for the *z*‐coordinates conditioned on the planar locations of the points. We fit the model to samples taken from healthy subjects and subjects suffering from diabetic neuropathy. In both groups, after a hardcore radius, there is some attraction between the end points. However, the range and strength of attraction are not the same in the two groups. Performance of the model is evaluated by using a cylindrical version of Ripley's *K* function due to the anisotropic nature of the data. Our findings suggest that the proposed model is able to capture the 3D spatial structure of the end points.

## Lay description

Diabetic neuropathy is a serious complication which affects a lot of people suffering from diabetes. It causes nerve damage, resulting in a variety of unpleasant symptoms such as pain and loss of sensation. As there is no treatment able to completely cure the diabetic neuropathy, diagnosis at the earliest possible stage is important to slow down its progression. To develop better diagnostic tools, skin samples from patients suffering from diabetic neuropathy and healthy controls have been collected. From these samples, the locations, where nerves enter the epidermis, branch, and terminate, can be identified using confocal microscopy imaging and image analysis procedures. In this work, we focus on the changes in the spatial pattern of the nerve endings at the early stages of the neuropathy. As data, we have skin samples from the feet of healthy subjects and subjects with mild diabetic neuropathy.

To investigate potential differences between the nerve end patterns between healthy subjects and patients suffering from diabetic neuropathy, we developed spatial point process models for the three‐dimensional locations of the nerve endings given the locations of the points, where nerves branch for the first time, and the locations of the entry points. These models were fitted to the available data for both groups and the estimated model parameters were compared. The model was able to describe the arrangement of the nerve endings quite well. Our results indicate that the nerves grow and interact in a different way in diabetic neuropathy and healthy samples. In particular, the clusters of the nerve endings in the diabetic neuropathy samples are tighter across the skin but further apart in the vertical direction compared to the clusters in the healthy patterns.

## 1 INTRODUCTION

Epidermal nerve fibres (ENFs) are thin sensory fibres in the epidermis, the outermost layer of the human skin. The ENFs are tree‐like structured nerves that grow and branch within the epidermis until they terminate. The termination points of the ENFs play an important role since they sense, for example, pain and heat and transfer then the signals to the brain. Peripheral neuropathy is a condition associated with poor nerve functionality and as the neuropathy progresses, the ENFs are damaged causing pain and loss of sensation. Here, we concentrate on diabetic neuropathy. Several studies^1^, ^2^, ^3^, ^4^ conclude that the ENF counts as well as the dermal and ENF coverage are reduced as the neuropathy progresses. It is also well established, that the 2D ENF patterns of subjects with diabetic neuropathy tend to be more clustered than healthy patterns.^2^, ^5^, ^6^, ^7^

The spatial structure of 2D locations of the end points, that is, the points where the nerve fibres terminate, has been investigated in earlier studies by regarding the locations of the points as realizations of spatial point processes. Since it is important to detect the neuropathy as early as possible, special attention has been paid to comparing healthy ENF patterns and patterns from subjects suffering from mild diabetic neuropathy both in terms of summary statistics and in terms of models. In the so‐called non‐orphan cluster (NOC) model^7^ and the uniform cluster centre (UCC) model,^2^ end point locations are constructed by conditioning on the observed base point patterns, which are the points where the nerve trees enter the epidermis. The major difference between the two models is the choice of the direction of the clusters with respect to the base points. In the NOC model, end point clusters favour directions towards open space, that is, away from the closest other base point, while in the UCC model no specific direction was preferred. For data from thighs from four healthy subjects, Garcia et al.^8^ introduced a continuous time birth and death process, where there is interaction between the base points and within the points in each end point cluster. Furthermore, Ghorbanbour et al.^3^ modelled interaction between the entire nerve trees, each consisting of an ENF base point and the end points connected to it, by a sequential marked point process model. Planar point process models for the spatial structure of the base points can also be found in the literature.^9^, ^10^

In the first 3D model proposed for the ENF structure, the first branching points were included.^11^ Given the observed base points, a model similar to the NOC model was suggested for the first branching points and then, a cluster model for the end points given the first branching points. However, this model was not able to describe the 3D ENF structure very well.

In this paper, our main objective is to construct a 3D point process model which takes into account interactions between the end points of the nerve fibres and can describe the complete ENF structure. For this purpose, we propose a model that allows interaction, attraction or repulsion, between pairs of end points. First, we model the planar locations of the end points by the two‐step NOC model introduced in Ref. 11 and then, given the planar coordinates, the third coordinate is modelled by a pairwise interaction Markov random field using cylindrical interaction regions as in Ref. 12. Since the end point patterns cannot be assumed to be isotropic, we use the cylindrical *K* function estimated in the three coordinate axis directions when describing the 3D spatial structure of the points.^13^, ^14^

The model is fitted to ENF samples taken from the feet of healthy controls and of subjects suffering from mild diabetic neuropathy. This comparison is particularly interesting since if detected early, it might be possible to slow down the progression of the neuropathy. We have some samples available also from patients suffering from later stages of the neuropathy which are not included in this study. These samples have only a small number of end points, not clusters of end points as in the healthy and mild neuropathy samples, and therefore, the model we will suggest here is not really suitable for such patterns. In addition, the nerve count alone is often enough to detect the later stages of the neuropathy. Our main finding was that the clusters in the mild neuropathy patterns tend to be tighter in the xy‐direction but the end points are further apart in the *z*‐direction compared to the clusters in the healthy patterns.

The paper is organized as follows. In Section 2, we describe the ENF data set. A brief introduction to point processes and point process summary statistics, including the cylindrical *K* function, is given in Section 3. In Section 4, we recall the definition of the two‐step NOC model and describe the pairwise interaction model for the *z*‐coordinates of the end points. Our results for the ENF data are presented in Section 5 and further discussed in Section 6.

## 2 DATA

The ENF data used in this study consist of skin samples taken from 32 healthy volunteers and eight patients suffering from mild diabetic neuropathy and were collected by Dr. Kennedy's group at the University of Minnesota.^15^, ^16^ Suction induced skin biopsies, in which a piece of the epidermis is removed, mounted on a slide and stained for confocal microscopy imaging, are used to acquire three to six samples from each patient. The nerves are then manually traced using Neurolucida software (microBrightField, Inc.) and the locations of the points, where the points enter the epidermis (base points), branch (branching points) and terminate (end points) are recorded.^15^, ^16^ Regarding the branching points, only the locations of the first branching points, that is, the points where the nerve fibres branch for the first time, are considered. For the remainder of this paper, branching points will refer to the first branching points.

**FIGURE 1:**
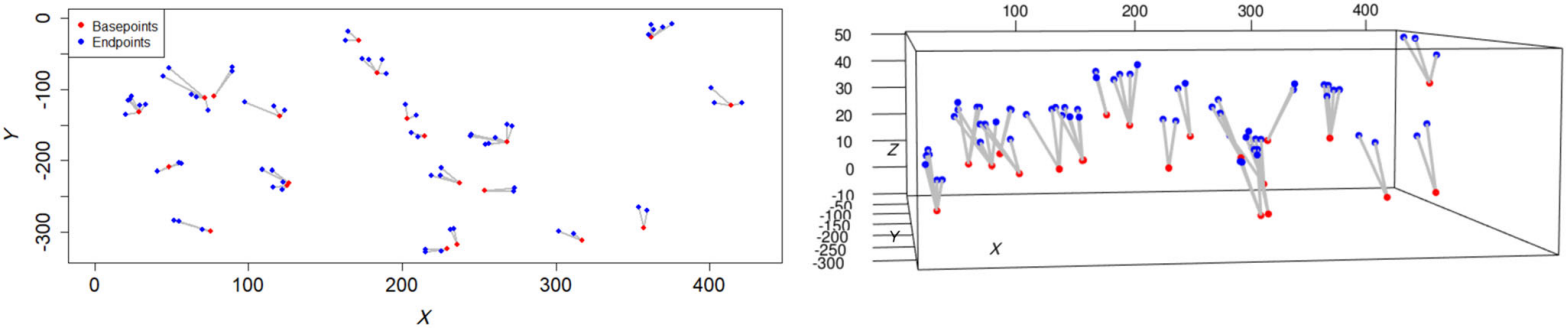
Structure of the nerve trees in 2D (left) and in 3D (right). Blue points correspond to the locations where the nerve fibres terminate and red points to the corresponding base points.

**FIGURE A.1:**
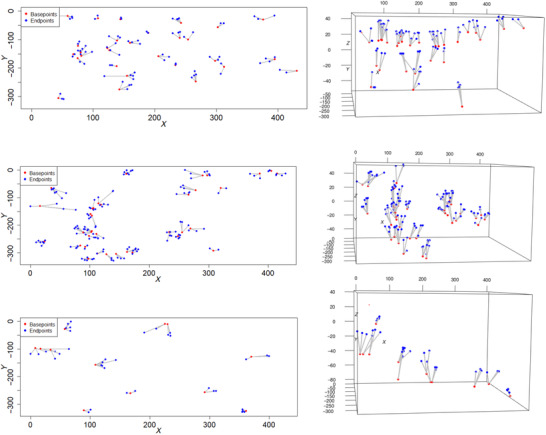
Structure of the nerve trees in 2D (left) and in 3D (right). Blue points correspond to the locations where the nerve fibres terminate and red points to the corresponding base points. The first point pattern is a healthy pattern and the other two patterns are mild diabetic patterns.

The data are in 3D and the samples are in boxes of size 320μm × 432 μm × *z* where *z* varies from 50 to 200 μm depending on the local thickness of the epidermis. For each subject, samples from six different body parts are available. As in some earlier studies,^2^, ^11^ we concentrate on early changes due to the neuropathy and compare data obtained from skin samples from the feet of 112 healthy samples and 28 samples from patients with mild diabetic neuropathy. Also, non‐spatial covariates such as age, gender and BMI for every subject are available, and the effect of these covariates on the spatial structure of ENF patterns taken from feet and calves of the subjects has been investigated using the same data set.^6^, ^17^ The conclusion was that the spatial pattern was affected by the covariates in the data from calf but not in the data from foot. In this study, we concentrate on the analysis of the observed spatial patterns alone. An example of an ENF sample is illustrated in Figure 1 (more samples are shown in Figure A.1 in the Appendix). Only the locations of the base points and end points are shown.

## 3 METHODS FOR SPATIAL POINT PROCESSES

The locations of the base, branching and end points of ENFs are regarded as realizations of spatial point processes. In this section, we briefly present some basic theory of spatial point processes. For more rigorous treatment of the subject, the reader is referred, for example, to Illian et al.,^18^ Diggle,^19^ Møller and Waagepetersen^20^ and Chiu et al.^21^ The definitions and notations given here mainly follow the book by Illian et al.^18^

### 3.1 Spatial point processes

Spatial point processes are mathematical tools used to study the arrangement of a set of locations where some phenomena of interest, such as locations of base points, end points or branching points, occurred. Even though the point process is defined in the whole spatial domain $D\subset R^{d}$, *d* being either 2 or 3 in our case, we usually observe a realization of the point process *X* in an observational window or box W⊂D. A point process *X* is simple, if at any location there is at most one point of the process, and *X* is locally finite, if for any bounded set *A* in the Borel set $B(R^{d})$, the process places a finite number of points in *A*. Moreover, a point process *X* is stationary if it is invariant under translations and isotropic if it is invariant under rotations about the origin. The point patterns of the ENFs are assumed to be realizations of simple, locally finite and stationary point processes.

### 3.2 Ripley's *K* function

Ripley's *K* function is a second‐order summary statistic of point processes which was originally defined for stationary and isotropic point processes. It is defined as the mean number of further points of the process in the *d*‐dimensional ball with radius *r* centred in an arbitrary point of the process divided by the intensity of the process (the mean number of points per unit volume). An estimator for the K(r) function needs to be edge corrected, as the points of the process outside the observation window are not taken into account into the estimation, making uncorrected estimators biased. The translation‐corrected estimator of the *K* function is given by

(1) $$\hat{K}\left(r\right)=\frac{1}{\hat{\lambda }n}\sum_{x_{1},x_{2}\in X\cap W}^{\neq }\frac{1}{|W\cap W_{x_{2}-x_{1}}|}1\left\{x_{1}-x_{2}\in B\left(o,r\right)\right\},\;r\geq 0,$$

where the ≠ above the summation sign denotes the summation over all distinct pairs, B(o,r) is a ball centred at the origin with radius *r* and $W_{x_{2}-x_{1}}$ is the window *W* translated by $x_{2}-x_{1}$. Furthermore, *n* is the total number of the observed points in *W*, ∣·∣ is the Lebesgue measure and $\hat{\lambda }$ is an estimator of the intensity.

Due to its symmetric structuring element, that is a 2D disc or a 3D ball, the isotropic *K* function is not an appropriate summary statistic for anisotropic point patterns. Directional *K* functions with different structuring elements have been recently developed as extensions of Ripley's *K* function for anisotropic point processes.^22^ Here, we recall the cylindrical *K* function, $K_{cyl}^{u}\left(r\right)$,^23^ which, as the name indicates, has a cylindrical structuring element. Multiplied by the intensity of the process, it gives the mean number of further points of the process within distance *r* from an arbitrary point *x* of the process that are inside the cylinder with some specified half‐width *w* directed towards *u* centred at *x*. Typically in the anisotropic case, the *K* function is estimated in different directions by orienting the cylinder accordingly. A translation edge‐corrected estimator for the cylindrical *K* function in the direction *u* is

(2) $${\hat{K}}_{cyl}^{u}\left(r\right)=\frac{1}{\hat{\lambda^{2}}}\sum_{x_{1},x_{2}\in X\cap W}^{\neq }\frac{1}{|W\cap W_{x_{2}-x_{1}}|}1\left[x_{1}-x_{2}\in B^{u}\left(r,w\right)\right],\;r>0,$$

where $\hat{\lambda^{2}}=\frac{n(n-1)}{|W{|}^{2}}$ and $B^{u}\left(r,w\right)$ denotes the shape created by the intersection of a cylinder with fixed half‐width *w* and direction *u* with a sphere of radius r>0. Note that above, we fix the half‐width *w* and define the cylindrical *K* function as a function of distance *r* only.

The variance stabilized and centred variant of Ripley's *K* function is defined by

(3) $$L\left(r\right)-r=\sqrt[{d}]{\frac{K(r)}{b_{d}}}-r,\;r>0,$$

where $b_{d}$ is the volume of the unit sphere in $R^{d}$. A similar transformed and centred variant of the cylindrical *K* function in $R^{3}$ is given by

(4) $$\begin{array}{ccc} & & L_{cyl}^{u}\left(r\right)-r=\frac{K_{cyl}^{u}\left(r\right)}{2\pi w^{2}}-r,\;r>0. \end{array}$$

These versions of the *K* functions are convenient since they equal 0 if the process is completely spatially random, that is, a homogeneous Poisson process. Positive values indicate clustering and negative regularity.

The summary functions described above can be used to characterize the spatial structure of one point pattern. When replicated data are available, we first estimate a summary function for each sample and then, pool the estimates together to obtain an estimate for the whole group. Our data are hierarchical, as samples from different subjects and subjects from different disease groups are available. Subjectwise summary functions can be obtained as a weighted average of the individual *K* functions, which are estimated from the different samples of the subject. In a similar manner, groupwise summary functions can be estimated as weighted averages of the subjectwise estimates. A more detailed discussion regarding this pooling procedure can be found in the Appendix. The cylindrical *K* function was estimated using the Kdirectional package in R.^24^

## 4 MODELLING ENF SPATIAL STRUCTURE

Below, we first recall the planar point process model for the projected end point locations introduced in Ref. 11 and then, suggest how to construct the *z*‐coordinates by using a Markov random field model.

### 4.1 Model for the planar point process $X_{p}$

Even though the model introduced in Ref. 11 was not able to completely capture the 3D structure of the ENF patterns, its 2D version turned out to be a good model for the 2D projections of the data. In this section, we review the 2D version of the model. It consists of two steps where in the first step the branching points are simulated conditioned on a realization of the base point patterns. In the second step, the end point clusters are simulated conditioned on the branching points constructed in the first step. As the model targets the whole nerve tree structure both branching and end points are modelled given the observed base points.

The model components in the first step of the model consist of the distance between the base points and the branching points *L*_1_ and the planar angle Φ_1_ of the segment connecting those points. The angle Φ_1_ is constructed to favour directions towards open space, that is the direction away from the closest other base point. This construction is similar to the construction of the NOC model presented in Ref. 7.

In the second step, the end point clusters are constructed around the simulated branching points. In this step, we not only have the distance between the end points and their branching point *L*_2_ and the direction Φ_2_ of the corresponding segments, but also the tree size *S* as in Ref. 2, that is the number of end points per cluster. The distributions of the different components of the model for the planar coordinates $X_{p}$ are the following:

1. Branching points | basepoints
$L_{1}∼Gamma\left(\alpha_{1},\beta_{1}\right)$
$\Phi_{1}∼VonMises\left(m,\kappa \right)$, where *m* is known.
2. Endpoints | branching points
$L_{2}∼Gamma\left(\alpha_{2},\beta_{2}\right)$
$\Phi_{2}∼Uniform\left(0,2\pi \right)$
S−1∼NB(s,p)

with NB denoting a negative binomial distribution. Parameters α_1_ and α_2_ denote the shape parameters, which control the shape of the respective Gamma distributions for the length of the two segments. Similarly, β_1_ and β_2_ denote the scale parameters, controlling the spread of the respective Gamma distributions. The concentration parameter κ of the von Mises distribution measures the concentration of the distribution around the mean direction *m*, where *m* is the direction away from the nearest other base point. The case when κ=0 corresponds to small concentration (large variance) and is equivalent to the uniform angular distribution while as κ increases, the distribution becomes more concentrated around the mean direction *m*. Finally, the parameters *s* and *p* of the NB distribution used for the tree size denote the number of successes and probability of success in a sequence of independent Bernoulli trials. We are mainly interested in the mean of the distribution, namely, $\mu =\frac{ps}{1-p}$.

To simplify the parameter estimation procedure, we assume as in Refs. 2, 7, 11, that all individual parts of the model are independent, hence parameter estimation can be performed independently for each component. The parameters of the Gamma and the NB distributions were estimated using maximum likelihood. Furthermore, since the mean directions *m* in the von Mises distributions are known, we used the simple approximation of the maximum likelihood estimate for κ proposed in Ref. 25.

### 4.2 Model for the *z*‐coordinates $X_{z}$ conditioned on $X_{p}$

**FIGURE 2:**
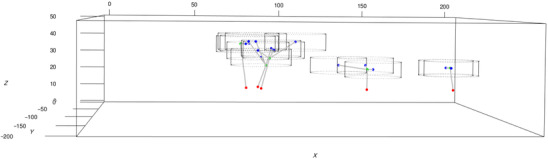
Example of the cylindrical interaction regions centred at the end points (blue dots) with parameters (w,2t)=(15,8). The locations of the base points (red dots) and the locations of the branching points (green dots) as well as the connections between them are shown.

Following Ref. 12, we propose a model for $X_{z}$ conditioned on a realization of $X_{p}$ defined by the conditional probability density

(5) $$\begin{array}{ccc} & & f\left({\left(z_{i}\right)}_{i=1}^{n}|{\left(x_{i},y_{i}\right)}_{i=1}^{n}\right)\propto \gamma^{s_{B_{r,t}\left({\left(z_{i}\right)}_{i=1}^{n}|{\left(x_{i},y_{i}\right)}_{i=1}^{n}\right)}}\\ & & \times \;1\left(\left\|\left(x_{i},y_{i},z_{i}\right)-\left(x_{j},y_{j},z_{j}\right)\right\|>h\;\text{for}\;1\leq i<j\leq n\right), \end{array}$$

where h>0 denotes the hard core distance, that is the minimum allowed distance between two points, and γ>0 denotes the interaction parameter. If there is no interaction between the points, γ=1. As the exponent of the interaction parameter is a non‐negative integer, values larger than 1 indicate attraction and values smaller than 1 repulsion between the neighbouring points in *X*. Moreover,

(6) $$s_{B_{r,t}\left({\left(z_{i}\right)}_{i=1}^{n}|{\left(x_{i},y_{i}\right)}_{i=1}^{n}\right)}=\sum_{1\leq i<j\leq n}1\left(\left(x_{i},y_{i},z_{i}\right)\in B\left(x_{j},y_{j},z_{j};\theta \right)\right)$$

is the number of neighbouring points in the interaction region $B(x_{j},y_{j},z_{j};\theta )$ which in our case is a cylinder centred at $\left(x_{j},y_{j},z_{j}\right)$ with radius and height parameters given by θ=(w,2t). To further illustrate this choice, an example of the shape of the end point clusters in 3D together with cylinders centred at the end points is displayed in Figure 2.

As defined above, the model for $X_{z}$ given the planar end point process $X_{p}$ is a pairwise interaction Markov random field, where two points $w_{1},w_{2}$ are neighbours if and only if $w_{1}\in B\left(w_{2};w,t\right)$ or if $∥w_{1}-w_{2}∥\leq h$ (in which case the conditional probability is equal to zero). Hence, the conditional density of $z_{i}$ given ${\left(x_{j},y_{j}\right)}_{i=1}^{n}$ and all other ${\left(z_{j}\right)}_{j\neq i}$ depends only on the neighbouring points of $\left(x_{i},y_{i},z_{i}\right)$. In particular, the conditional density of $z_{i}$ given all other $z_{j}$, j≠i, and a realization of the planar point process $X_{p}$ is given by

(7) $$\begin{array}{ccc} & & f\left(z_{i}|{\left(x_{k},y_{k}\right)}_{k=1}^{n},{\left(z_{j}\right)}_{j\neq i}\right)\propto \gamma^{s_{i}}1\\ & & \left(\left\|\left(x_{i},y_{i},z_{i}\right)-\left(x_{j},y_{j},z_{j}\right)\right\|>h\;\text{for}\;j\neq i\right), \end{array}$$

where

(8) $$s_{i}=\sum_{j:j\neq i}1\left(\left(x_{j},y_{j},z_{j}\right)\in B\left(x_{i},y_{i},z_{i};\theta \right)\right).$$

### 4.3 Parameter estimation

To estimate the parameters Φ=(γ,h,w,t) of the model (6), we first estimated the hard core distance *h* by $\hat{h}=\left(n-1\right)d_{min}/n$, where $d_{min}$ is the observed minimum distance between two points. Then, the remaining parameters were estimated by combining pseudo‐likelihood estimation of γ and grid search of the parameters *w* and *t*. We maximize the log pseudo‐likelihood given by

(9) $$\begin{array}{c} pl\left(\gamma ,h,w,t\right)=\sum_{i=1}^{n}\log\left(f(z_{i}|{\left(x_{j},y_{j}\right)}_{j=1}^{n},{\left(z_{j}\right)}_{j\neq i}\right)\\ =\sum_{i=1}^{n}\log\left(\gamma^{s_{i}}1\left(\left\|\left(x_{i},y_{i},z_{i}\right)-\left(x_{j},y_{j},z_{j}\right)\right\|>h\;\text{for}\;j\neq i\right)/c_{i}\right), \end{array}$$

where $c_{i}$ is the normalizing constant defined as

(10) $$\begin{array}{c} c_{i}=\sum_{k=0}^{n-1}\gamma^{k}\int_{W_{z}}1\left(\left\|\left(x_{i},y_{i},z\right)-\left(x_{j},y_{j},z_{j}\right)\right\|>h\;\text{for}\;j\neq i\right)\\ \times 1\left(\sum_{j\neq i}1\left(\left(x_{j},y_{j},z_{j}\right)\in B\left(x_{i},y_{i},z;w,t\right)\right)=k\right)dz, \end{array}$$

with respect to γ where $W_{z}=\left[0,Z_{max}\right]$ is the window in the *z*‐direction. More specifically, numerical optimization was used to maximize the pseudo‐likelihood $pl(\gamma ,\hat{h},w_{i},t_{i})$ over a grid of values for the parameters $w_{i}$ and $t_{i}$ that define the region of interaction. In other words, $\hat{\gamma }$ is the parameter estimate for γ that gives the highest pseudo‐likelihood value and the corresponding grid values give estimates $\hat{w}$ and $\hat{t}$ for *w* and *t*, respectively. Minus sampling was used to reduce the bias in the parameter estimates caused by edge effects.

### 4.4 Simulation

To simulate a 3D point pattern from the model, we initially choose a model that can produce reasonable realizations of the planar point process $X_{p}$ model described in Section 4.1. Simulating from this planar model is a two‐step procedure. First, conditioned on an observed point pattern for the base point locations, the directions of the branching points are calculated from the data and the branching points are simulated given the distribution for the branch length. Then, the end point clusters are constructed around the simulated branching points.

To obtain a realization from the full model, we then conditioned on a simulated planar pattern $X_{p}$ and then, simulated the 1D point process using a Metropolis–Hastings algorithm, where the number of points in $X_{p}$ is fixed (algorithm 7.1 in Ref. 20). Moreover, properties of the Markov chain created by the specific Markov chain Monte Carlo (MCMC) algorithm, such as irreducibility and reversibility, are proved in proposition 7.11 in Ref. 20.

Let us assume without any loss of generality that $X_{p}$ consists of *n* points. For each point in $X_{p}$, we initialize its *z*‐coordinate as a random location in $W_{z}$. Then, in every iteration of the algorithm, we cycle through every point in $X_{p}$ and propose a new point $z_{i}^{new}$ in $W_{z}$ using a uniform proposal. The new proposed point is accepted with probability

(11) $$\alpha =\frac{f(z_{i}^{new}|X_{p},{\left(z_{j}\right)}_{j\neq i})}{f(z_{i}|X_{p},{\left(z_{j}\right)}_{j\neq i})},$$

where *f* is the conditional density defined in Equation (7). The algorithm used to simulate the $X_{z}$ given $X_{p}$ is displayed in Algorithm 1.

Algorithm 1

  

**Table:** 

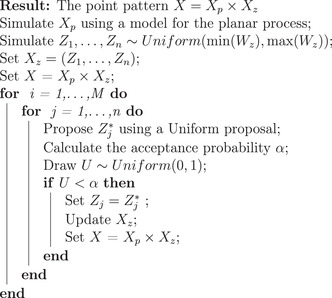

## 5 MODELLING THE NERVE FIBRE DATA

Below, we fit the model introduced in Section 4 to each subject in the healthy and mild groups. The subjectwise estimates for the model parameters are obtained by using all the samples available for that subject. The spatial structure of the end points in the healthy and mild groups is compared in terms of the parameter estimates and the pooled cylindrical *K* functions.

### 5.1 Model for the planar process $X_{p}$

**FIGURE 3:**
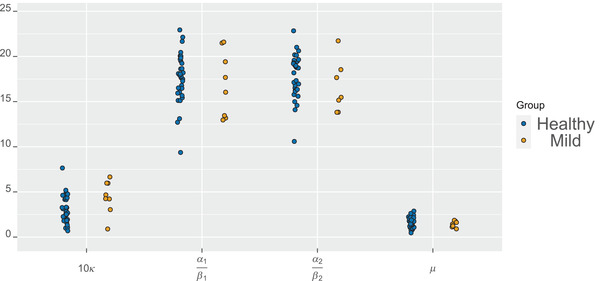
Dotplot for the parameter estimates of the 2D non‐orphan cluster (NOC)‐like model for all the subjects in the two groups: concentration parameter κ of the von Mises distribution, means $\alpha_{1}/\beta_{1}$ and $\alpha_{2}/\beta_{2}$ of the Gamma distributions representing the segment lengths base point–branching point and branching point–end point, respectively, and the mean μ of the NB distribution representing the number of end points per cluster.

First, we fit the two‐step model for the planar process $X_{p}$. The subjectwise parameter estimates are illustrated in Figure 3. The estimates for the branch length and concentration parameter of the von Mises distribution differ in the two groups. In particular, the branches are longer and the concentration parameters smaller in the healthy group, indicating smaller clusters (in area) and less concentration of the segment directions around the mean direction than in the mild group. Moreover, the number of end points per cluster tends to be smaller in the mild group than in the healthy group.

### 5.2 Model for the 3D structure

#### 5.2.1 Parameter estimates

**FIGURE 4:**
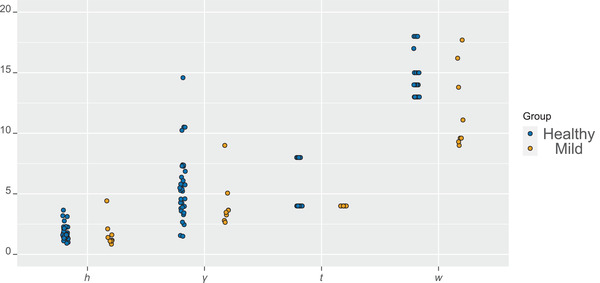
Parameter estimates of the hardcore distance *h*, the interaction parameter γ, and the optimal cylindrical parameters *t* and *w* for the two groups

The parameters of the Markov field model are estimated using the methodology described in Section 4.3 and a boxplot of the estimates is given in Figure 4. The interaction volumes (cylinders) are wider in the healthy group compared to the mild group. Outside the hardcore distance, there is some attraction (γ>1) between the end points in both groups. The hard core distance *h* and cylinder height *t* are estimated quite accurately. However, there is quite a lot of variation in the estimates of the interaction parameter γ in the healthy case and the cylinder width in the mild case. The half‐width parameter *w* and the interaction parameter γ are highly correlated which would suggest to fix *w* to a constant value. However, fixing *w* did not affect the estimates of γ a lot, and we, therefore, kept the individual estimates. We also identified some outlier samples, which have significantly smaller end point intensities and larger hardcore distances than the other samples. The outlier samples were not included when the groupwise goodness of fit of the model was evaluated.

#### 5.2.2 Goodness of fit of the 3D model

**FIGURE 5:**
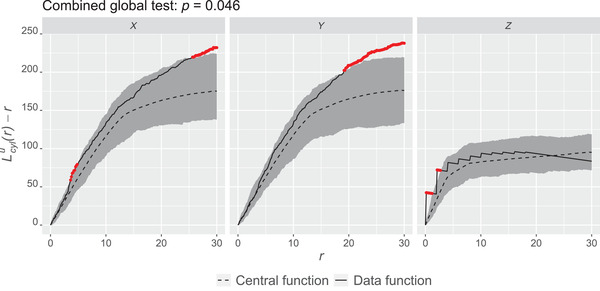
Groupwise pooled $L_{cyl}^{u}\left(r\right)-r$ functions with 95% global envelopes for the end points from the healthy samples in the *x*‐axis (left), *y*‐axis (middle) and *z*‐axis (right) directions

**FIGURE 6:**
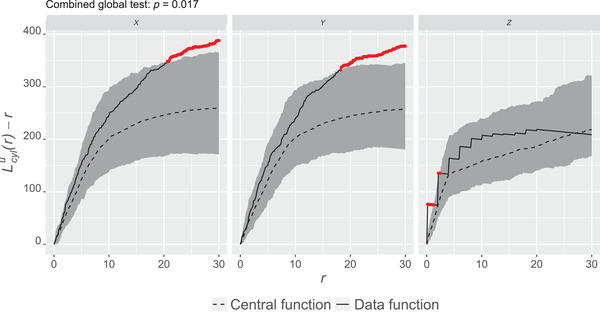
Groupwise pooled $L_{cyl}^{u}\left(r\right)-r$ functions with 95% global envelopes for the end points for the samples from patients with mild diabetic neuropathy in the *x*‐axis (left), *y*‐axis (middle) and *z*‐axis directions

**FIGURE A.2:**
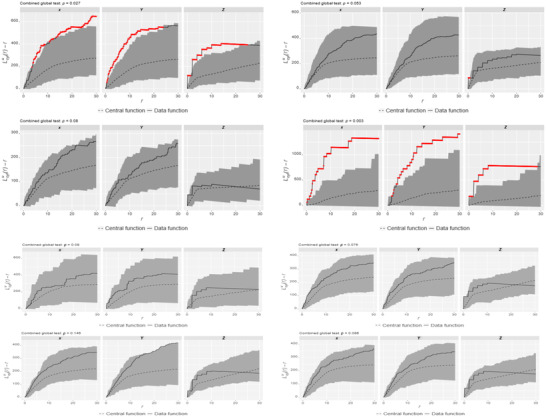
Subjectwise pooled cylindrical L(r)−r functions with global envelopes for the end points for every subject with mild diabetic neuropathy from the model

**FIGURE A.3:**
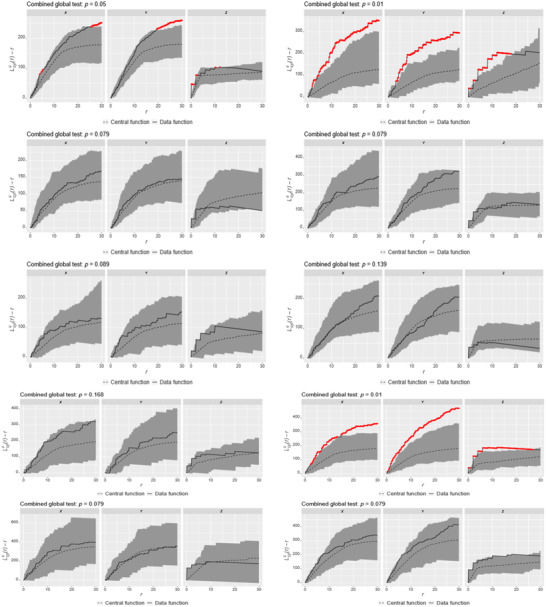
Subjectwise pooled cylindrical L(r)−r functions with global envelopes for the end points of 10 healthy subjects from the model

Goodness of fit of the model is evaluated in terms of groupwise pooled cylindrical $L_{cyl}^{u}\left(r\right)-r$ functions in the three coordinate axes directions using the fixed cylinder half‐width *w* equal to 7 μm. The half‐width of the cylinder was chosen to be smaller than the cluster radius. As can be seen in Figure 5, the model is able to capture the 3D spatial structure of the end points within the groups quite well even though the envelopes (based on 2500 simulations) do not completely cover the empirical curves. The corresponding results for the mild group are very similar (see Figure 6). The subjectwise models fit also quite well to the data in most cases (see Figure A.2 and Figure A.3 in the Appendix). We can also see in Figures  and 6 that the point patterns are not isotropic, especially in the healthy group, since the summary function curves in the *z*‐direction differ from the curves in the *x*‐ and *y*‐directions. The points seem to be in tighter clusters in the *z*‐direction compared to the other two since the maximum of the *L* function is reached at a shorter distance.

### 5.3 Groupwise comparisons

**FIGURE 7:**
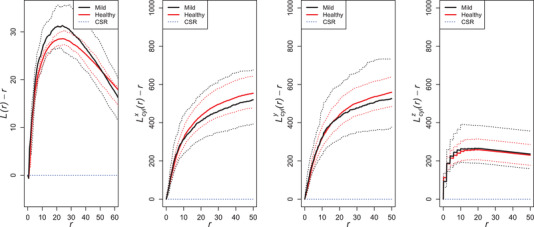
Groupwise pooled isotropic L(r)−r function (left) and groupwise pooled cylindrical $L_{cyl}^{u}\left(r\right)-r$ functions with cylinders directed towards the three axes, for the end points from the healthy group (red) and from the mild group (black) with 95% pointwise bootstrap envelopes (dashed lines). The theoretical value of the function under complete spatial randomness (CRS) is illustrated with a dashed blue line.

We first compared the 3D spatial structure of the end points in the two groups by plotting the estimated groupwise cylindrical $L_{cyl}^{u}\left(r\right)-r$ functions with fixed cylinder half‐width w=7μm directed towards the three coordinate axes. We clearly see in Figure 7 that the end point patterns are not isotropic. Furthermore, even though the mild patterns seem to be slightly more clustered than the healthy patterns, this difference is not statistically significant.

Some differences can, however, be seen in the parameter estimates of the model shown in in Figures 3 and 4. Branching points tend to be further away from their base points and angles more concentrated away from the nearest other base points in the mild group than in the healthy group. Also, there are less end points per cluster and the end points are closer to their branching points in the mild case compared to the healthy case. Concerning the Markov model parameters, the hard core distance seems to be larger, attraction stronger (γ larger), and cylinder width larger in the healthy group than in the mild group. However, the variation of the estimates is quite large.

## 6 DISCUSSION

We have treated the locations of the base, branching and end points of ENFs extracted from confocal microscopy images as realizations of multi‐type 3D point processes and proposed a point process model for the spatial structure of the nerve trees given the base points. The model is constructed in two steps. First, we constructed the planar coordinates of the end points using a planar point process model suggested in Ref. 11 and then, we constructed the *z*‐coordinates given the planar coordinates using a pairwise interaction Markov field model which allows interaction between the end points. Due to the anisotropy of the end point patterns, cylindrical interaction regions were chosen.

The underlying point process is assumed to be stationary. Some of the patterns look heterogeneous but since the samples are quite small, it is difficult to say whether they are heterogeneous or taken from a larger, homogeneous, area. Therefore, we think that the assumption of stationarity is reasonable. One possible cause for the heterogeneity of the base points could be the dermal papillae in the dermis, the layer below the epidermis, which forms ridges in the dermis and makes the thickness of the epidermis vary. However, we do not have any spatial information on the dermal papillae.

The model was fitted to the nerve patterns from healthy subjects and from subjects suffering from mild diabetic neuropathy. In both groups, there is some minimum interpoint distance between the end points but at slightly larger distances they attract each other. However, the attraction is weaker and the interaction range smaller in the neuropathy patterns compared to the healthy ones.

Recall that in the planar case, the end point patterns from patients suffering with diabetic neuropathy are clearly more clustered than the patterns from healthy volunteers,^2^, ^7^ particularly, the patterns in the mild diabetic group are more clustered than the patterns in the healthy group. The difference in clustering is not as clear in 3D. The *K* functions did not show any significant difference in clustering and the differences in the estimates of the model parameters were quite small in the two groups. This indicates that the end point clusters in mild neuropathy patterns tend to be tighter on the xy‐direction but the end points are further apart in the *z*‐direction compared to clusters in healthy patterns.

Having only small differences in 3D clustering between healthy and neuropathy patterns supports the idea that the depth of the end points does not affect their ability to sense (personal communication with Adam Loavenbruck, University of Minnesota). Therefore, when investigating the change in clustering of ENFs as neuropathy advances, our results indicate that it seems to be enough to study only the 2D ENF coverage across the skin and therefore, the results presented here do not provide any straightforward clinical implications. However, to understand the complete 3D structure of the ENFs, how the nerve fibres grow and interact with each other, and whether there are differences between healthy subjects and patients with diabetic neuropathy concerning these issues, a 3D model for the structure is necessary.

Even though the 3D model suggested in this paper fits the data quite well, it could be further improved. Instead of modelling the planar coordinates first, we could find a 3D model with interaction within and between base points, branching points and end points. For instance, the interaction cluster point process model introduced in Garcia et al.^8^ for the planar point patterns could be a good starting point. Furthermore, the 2D model for the branching points could be improved by taking into account not only the closest base point but all base point. Another interesting research question would be to model the alterations in the spatial structure of the nerve patterns as the neuropathy develops. As every diseased pattern is obtained from a healthy pattern by removing some of the nerve trees and/or end points, appropriate thinning strategies could be constructed to mimic the removal of parts of the nerve structure. Finally, given a good model for the ENF structure, the ENF data set could be augmented with simulated point patterns obtained by the model. Such data could then be used to train discrimination algorithms to determine the level of the neuropathy in a patient.

## APPENDIX A

### A.2 Pooling procedure of the summary functions

Our data are hierarchically structured into disease groups (healthy and patients with mild diabetic neuropathy), different subjects from those groups, and different samples from the subjects, and we are especially interested in comparing the spatial structure of the epidermal nerve fibres (ENFs) between the two groups. Given that estimates for the samplewise summary functions ${\hat{K}}_{ij}\left(r\right)$ of subject *i* and sample $j\in \{1,\text{…},m_{i}\}$ are available, we can estimate the subjectwise summary functions ${\bar{K}}_{i}\left(r\right)$ for subject *i* by

(A.1)
$${\bar{K}}_{i}\left(r\right)=\sum_{j=1}^{m_{i}}w_{ij}{\hat{K}}_{ij}\left(r\right),\;w_{ij}=\frac{n_{ij}^{2}}{\sum_{k=1}^{m_{i}}n_{ik}^{2}}.$$

Then, the estimates for the groupwise summary function ${\bar{K}}_{g}\left(r\right)$ are given by

(A.2)
$${\bar{K}}_{g}\left(r\right)=\sum_{i=1}^{N}w_{i}{\bar{K}}_{i}\left(r\right),\;w_{i}=\frac{n_{i}^{2}}{\sum_{k=1}^{N}n_{k}^{2}},$$

where $n_{ij}$ are the total number of points in sample *j* of subject *i* and $n_{i}=\sum_{k=1}^{m_{i}}n_{ik}$ are the total number of points in the samples of subject *i*. We used square point number weights as the patterns from different subjects, and the patterns from different subjects within a disease group cannot be assumed to have the same intensity.

### A.3 Global envelope tests

Graphical non‐parametric Monte‐Carlo tests for functional and multivariate statistics known as global envelope tests have recently been developed.^26^ Let ${\left\{T_{i}\right\}}_{i=1}^{s}$ be *s* discretized at *d* points functional statistics with **T**_1_ being the empirical statistic and ${\left\{T_{i}\right\}}_{i=2}^{s}$ the simulated statistics obtained under the null model. A global envelope is a band constructed by simulated statistics under the null model such that the probability that a statistic under the null model wanders outside the envelope in any of the *d* points is α. Hence, global envelope test are widely used to assess goodness of fit of spatial point process models.

The construction of global envelopes is based on the choice of a measure M which determines the α most extreme statistics obtained under the null model. Then minima and maxima of the less extreme statistics for each of the *d* points, are used to construct the envelope. In this work, the extreme rank length (ERL) measure was considered to obtain 95% global envelopes. For more rigorous description of the chosen measure, you are referred to Ref. 27.

Generalizations of the global envelopes, called combined global envelopes, have been developed for the case where we want to jointly assess the significance of *G* different statistics $T_{i}^{j}$ with j∈{1,…,G}. We note that the combined global envelope provides one *p*‐value for the test but constructs *G* different global envelopes, one for each test statistic. In this work, global envelope tests were used to test the hypotheses that cylindrical *L* functions directed towards the three main axes obtained from the model are the same as the empirical corresponding summary statistics.
